# Supplementary material for: Causal interplay between lactose intolerance and gut microbiota: a combined bidirectional Mendelian randomization and in vivo validation study
Source: Front Nutr. 2026 Jun 1;13:1803337. doi: 10.3389/fnut.2026.1803337 (PMC13265576; doi:10.3389/fnut.2026.1803337)
Supplement: Supplementary file 1 [file Data_Sheet_1.zip › supplementary materials/Forward/leave one out cross validation/ebi-a-GCST90027600.finngen_R12_E4_LACTONAS.pdf]

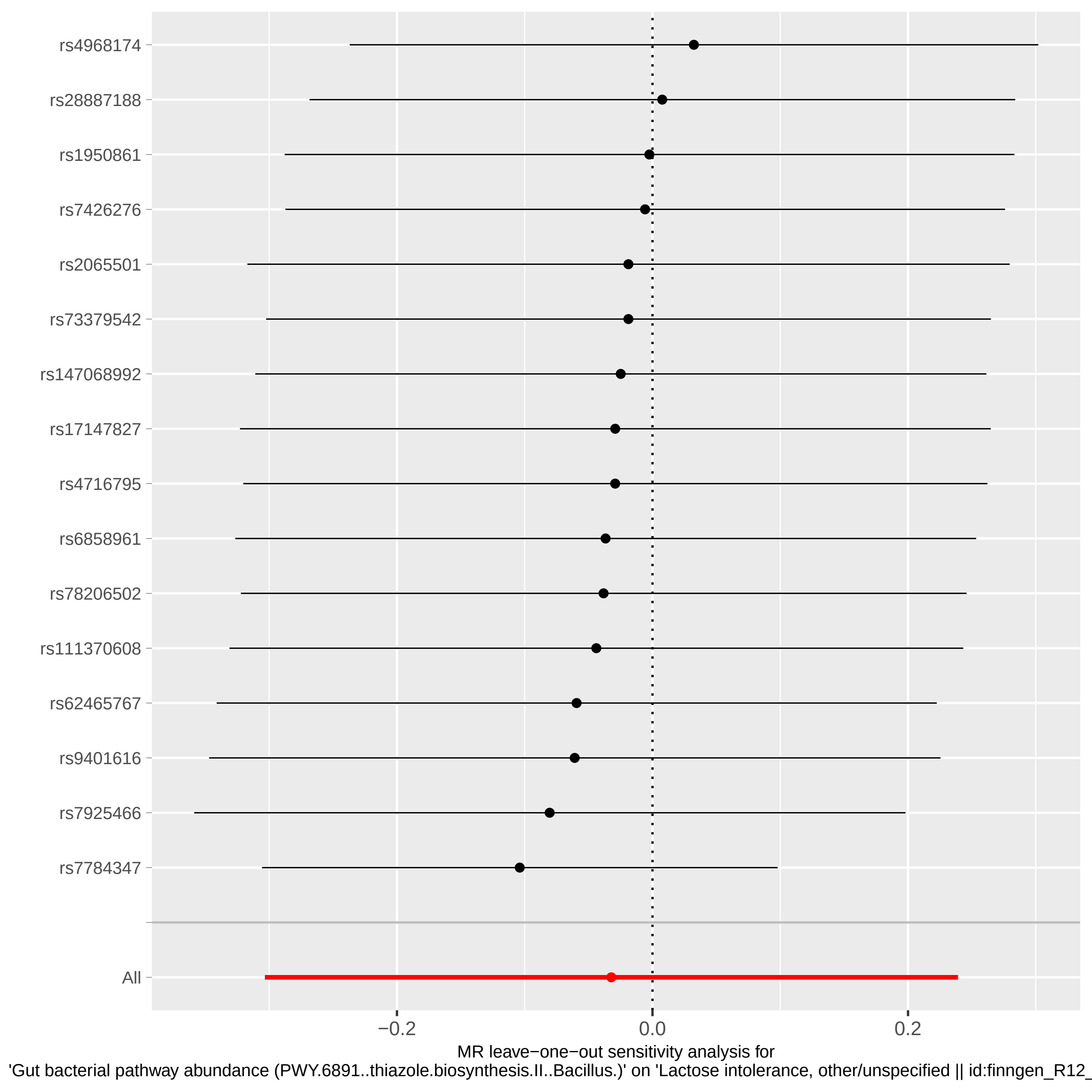
rs4968174
rs28887188
rs1950861
rs7426276
rs2065501
rs73379542
rs147068992
rs17147827
rs4716795
rs6858961
rs78206502
rs111370608
rs62465767
rs9401616
rs7925466
rs7784347
All
−0.2
0.0
0.2
MR leave−one−out sensitivity analysis for
'Gut bacterial pathway abundance (PWY.6891..thiazole.biosynthesis.II..Bacillus.)' on 'Lactose intolerance, other/unspecified || id:finngen_R12_
